# Flipped Classroom versus Traditional Didactic Classroom in Medical Teaching: A Comparative Study

**DOI:** 10.7759/cureus.23657

**Published:** 2022-03-30

**Authors:** Milav H Bhavsar, Hardik N Javia, Sanjay J Mehta

**Affiliations:** 1 Biochemistry, C.U. Shah Medical College, Surendranagar, IND; 2 Biochemistry, C. U. Shah Medical College, Surendranagar, IND; 3 Microbiology, C.U. Shah Medical College, Surendranagar, IND

**Keywords:** traditional didactic classroom, mbbs, biochemistry, flipped classroom, medical education

## Abstract

Background

The effective and efficient delivery of healthcare services that the National Medical Commission (NMC), India desires from Indian Medical Graduate (IMG) can only be fulfilled if the learner’s participation is extensive. Flipped classroom (FC) may promote enhanced as well as meaningful learning and critical thinking in students. By implementing this method trend can be changed from a teacher-centered approach to a student-centered approach, thus teaching-learning becomes more effective and interesting. It promotes learning and thinking helping the students in diagnosis and formulating appropriate management of patients during the clinical years of medical teaching of MBBS (Bachelor of Medicine and Bachelor of Surgery) and thereafter.

Aim

To compare FC and traditional didactic classroom (TDC) teaching for first-year MBBS students.

Objectives

-To evaluate FC method teaching for first-year MBBS students.

-To compare effectiveness of FC and TDC teaching for first-year MBBS students.

-To evaluate perception of students toward FC teaching method.

Methodology

The study was conducted after getting approval from the Institutional Ethics Committee. Total of 100 students volunteered to participate in the study after providing informed consent. Two groups based on pseudo randomization were created and subjected to the FC method and TDC method in module A and crossover of groups was done in module B. Both groups were subjected to post-test after intervention in modules. Feedback was obtained from students on their perception toward the FC method.

Results

There was a statistically significant difference (p<0.05) in post-test scores of both FC groups in both modules (FC Method: 14.77 ± 2.16 and 11.26 ± 1.76 vs TDC Method: 12.16 ± 2.05 and 10.03 ± 2.57). Overall positive feedback was received for FC method of teaching compared to TDC method.

Conclusions

Considering responses and results of the assessment, it can be concluded that the FC approach is beneficial for students. It enhances the learning of students. Perception of students toward medical teaching can be greatly improved. It helps students achieve better results in their learning. With larger sample size studies, this result of FC method being a better learning tool will gather more strength.

## Introduction

Medical students may find it difficult to correlate knowledge of biochemistry with clinical conditions and hence the effective and efficient delivery of healthcare services that the National Medical Commission (NMC) desires from IMG are not fulfilled completely [1]. The introduction of flipped classroom (FC), a recent method that requires learner’s participation to a very great extent may promote enhanced as well as meaningful learning and critical thinking in medical students. By implementing FC method, the trend can be changed from a teacher-centered approach to a student-centered approach, thus teaching-learning becomes more effective and interesting. The use of both traditional and innovative methods can be complementary to the learning process. This will promote meaningful enhanced interactive learning, critical thinking, and problem-based learning that will help the students in clinching diagnosis and formulating appropriate management of patients during the clinical years of MBBS and thereafter [2].

The effective and efficient delivery of healthcare services requires knowledge, technical, analytical, and communication skills. Healthcare professionals need to be trained adequately to fulfill this objective. The teaching-learning methods used in medical education should be able to achieve a higher level of Bloom’s Taxonomy of the cognitive domain. Fostering higher-order thinking among students for understanding a subject was a key point in the previous study [3]. Flipped learning is a pedagogical approach in which direct instruction moves from the group learning space to the individual learning space, and the resulting group space is transformed into a dynamic, interactive learning environment where the educator guides students as they apply concepts and engage creatively in the subject matter [4]. This learner-centric model enables the educator to provide activities in the classroom that are action-based, authentic, connected, collaborative, innovative, high-level engagement, experience-based, project-based, inquiry-based, and self-actualizing [5]. FC method generates an environment that is learner-centered and imparts deep and critical thinking with meaningful as well as enhanced learning [6].

The current study aims to compare FC and TDC methods of teaching for first-year MBBS students. Objectives are to evaluate FC teaching method, to compare the effectiveness of FC and TDC methods, and to evaluate the perception of students toward FC teaching method.

## Materials and methods

Conducted study was primary, interventional, and academic (educational) in design. The study was conducted in the Department of Biochemistry, C.U. Shah Medical College (CUSMC), Surendranagar after approval from Institutional Ethics Committee - Human Research, CUSMC, Surendranagar, Gujarat, India with approval number CUSMC/IEC(HR)/RP/16/2021/Final Approval/79/2022.

Freshly joined first-year MBBS students (N=100) of the ongoing batch volunteered in the study. Other ongoing first-year MBBS students from previous batches were excluded. Written informed consent was obtained from all students before the study intervention. The maximum period of study was 6 months out of which intervention was 2-3 weeks. Two groups were created by pseudo randomization based on odd-even roll numbers. Each group was having 50 students. But not all students completed the duration of the study intervention and were absent in parts of teaching modules due to various reasons. Such students were excluded for study purposes. Final numbers for group 1 were 39 and for Group 2 were 43.

Module development

Two FC modules from core area topics of the biochemistry syllabus were developed according to standard guidelines and validated through peer review by subject experts. FC Topics were Vitamin B1 Deficiency (Module A) and Iron Deficiency: Anemia (Module B).

Project implementation

**Figure 1:**
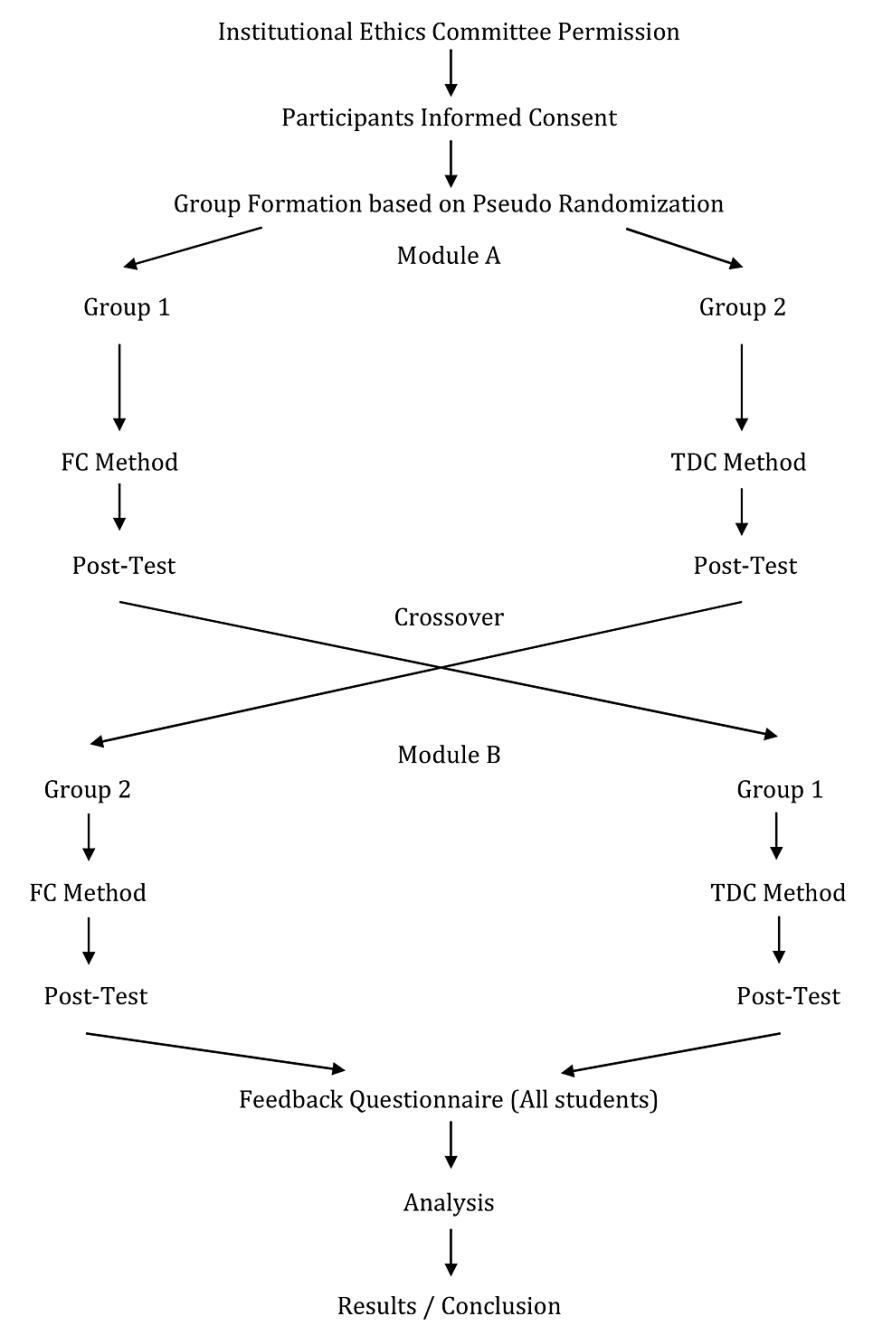
Study Outline FC: flipped classroom; TDC: traditional didactic classroom

One faculty conducted FC session in group 1 as per the method described in the outline (Figure 1) while the other faculty conducted the same session through TDC method in group 2 at the same time. FC session was implemented over a period of 1 week. Objective assessment (post-test) was done for both groups in modules A & B.

Module A: Vitamin B1 Deficiency (Table 1)

**Table 1:** Module Details

Pre-session: (6 days)	
Group 1	Group 2
The topic was introduced along with learning objectives. The group was included in Institutional Google Classroom platform for learning resource sharing. Resources were carefully selected based on learning objectives which included case scenarios, textbook excerpts, online videos, etc. related to topic. Group was asked to prepare the topic well in advance.	Only topic was introduced during pre-session. Group was not having any kind of resource sharing during pre-session.
Session: (7th day 60 minutes)	
Group 1	Group 2
Learning objectives were reinforced. As per flipped classroom method, students were asked to engage in discussion among themselves by throwing various questions. Session was facilitated by faculty and was concluded with narration and important points.	Topic was introduced along with learning objectives. Students were engaged through a regular didactic lecture.
Post-session Part 1 (Post-test): (10 minutes)	
Groups 1 & 2 Assessment: Students were subjected to post-test	
Post-session Part 2 (Feedback): (10 minutes)	
Feedback of students was obtained through questionnaires.	

Module B: Iron Deficiency Anemia

Module B was conducted the same as module A except for crossover of groups and faculties was done. Group 2 was subjected to FC method and group 1 was subjected to TDC method.

Feedback

The perception of students toward FC method of teaching was obtained through a pre-validated questionnaire after sessions. The questionnaire was based on a 5-point Likert scale (strongly agree, agree, neutral, disagree, and strongly disagree). The questionnaire included open-ended questions based on experience, outcome, resource material, methodology, etc. Internal consistency was calculated for the questionnaire (Cronbach’s alpha=0.825).

Statistical analysis

Analysis was done through SPSS (IBM, Chicago, IL, USA) software. Mean and standard deviation (SD) were used for descriptive analysis. Shapiro-Wilk test was used for normality. Chi-square test was applied for categorical variables. Continuous variables were analyzed using unpaired student’s t-test. Value of p<0.05 was considered significant.

## Results

**Figure 2:**
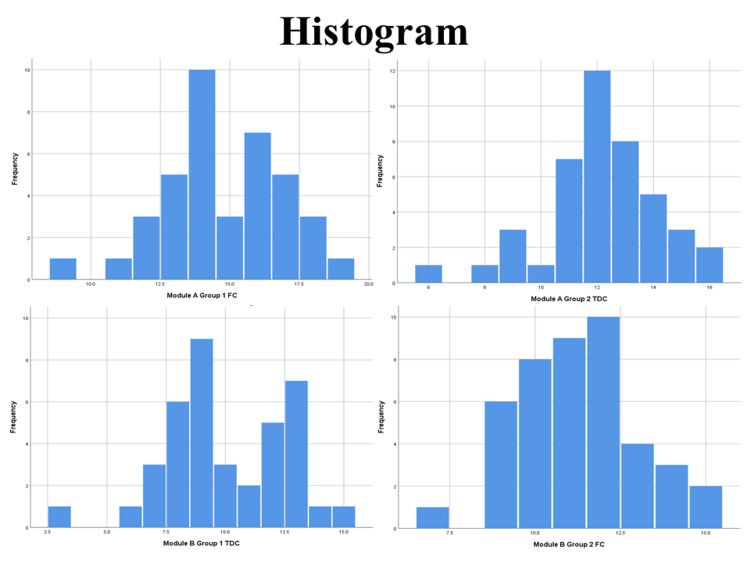
Histogram Chart for Normality FC: flipped classroom; TDC: traditional didactic classroom

Out of 100 enrolled students, 82 students completed all the phases of intervention during the study (response rate 82%). Group 1 had 39 students (78%) while group 2 had 43 students (86%). Both groups were subjected to both methods (FC and TDC) of learning via two separate modules implementing crossover between modules between sessions. Post-test results obtained were checked for normality using the Shapiro-Wilk test among all groups (Figure 2 and Table 2) and found to be normally distributed (p>0.05).

**Table 2:** Normality Test Results FC: flipped classroom; TDC: traditional didactic classroom; SD: standard deviation

Data	Module A		Module B	
	Group 1 (FC) (N=39)	Group 2 (TDC) (N=43)	Group 1 (TDC) (N=39)	Group 2 (FC) (N=43)
Mean	14.77	12.16	10.03	11.26
SD	2.16	2.05	2.57	1.76
Shapiro-Wilk	0.967	0.949	0.950	0.962
Significance	p=0.298	p=0.054	p=0.081	p=0.162

Post-test results were analyzed among groups per module by making three different result categories (<10 (failed), 10-13(passed), and >=14(excellent) out of 20). Results were found to be statistically significant (p<0.05) among students learning through FC method (Table 3).

**Table 3:** Categorical Post-test Scores Comparison FC: flipped classroom; TDC: traditional didactic classroom

Category	Module A		Module B	
	Group 1 (FC) (N=39)	Group 2 (TDC) (N=43)	Group 1 (TDC) (N=39)	Group 2 (FC) (N=43)
1 (<10)	1	5	20	7
2 (10-13)	9	28	17	30
3 (>=14)	29	10	2	6
Chi square	21.536		11.6877	
Significance	p=0.000021		p=0.002898	

The mean score of students who learned through FC method was higher compared to TDC method. Unpaired student’s t-test was applied to determine the significant difference between two groups (FC vs TDC) in each module. Results obtained were statistically significant (p<0.05) for each module in FC method of learning (Table 4).

**Table 4:** Post-test Scores Comparison FC: flipped classroom; TDC: traditional didactic classroom; SD: standard deviation

Data	Module A		Module B	
	Group 1 (FC) (N=39)	Group 2 (TDC) (N=43)	Group 1 (TDC) (N=39)	Group 2 (FC) (N=43)
Mean	14.77	12.16	10.03	11.26
SD	2.16	2.05	2.57	1.76
t-statistics	-5.613		2.549	
Significance	p<0.0001		p=0.0127	

**Figure 3:**
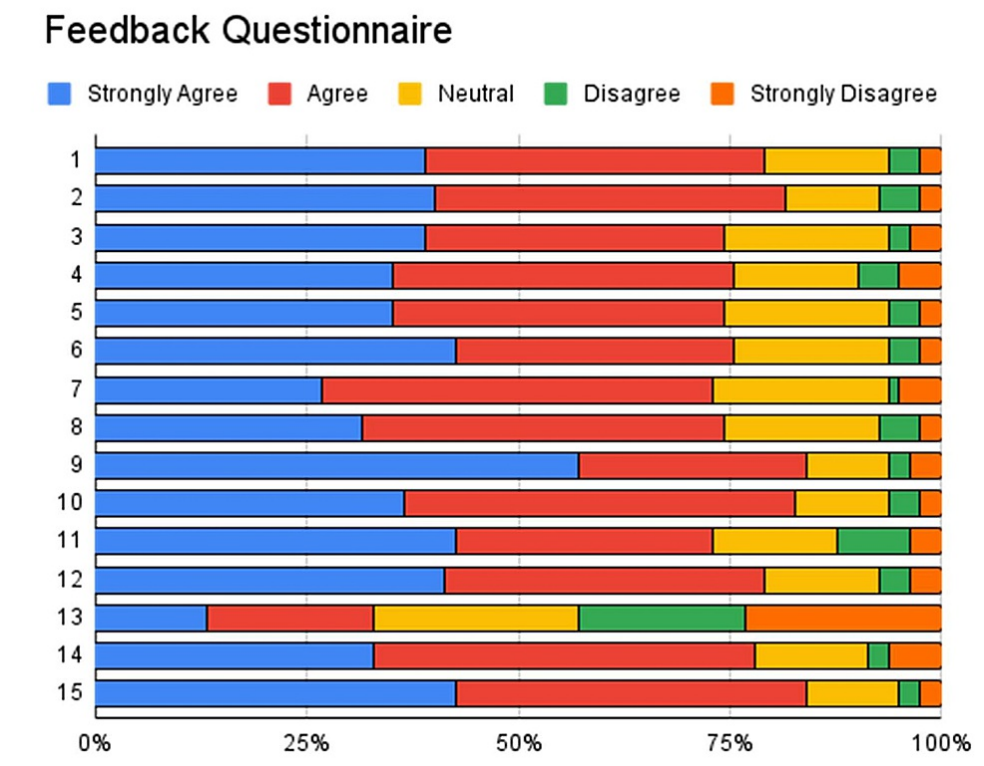
Feedback Questionnaire Visual Representation X axis: response percentage; Y axis: question numbers

Feedback from students regarding perception toward FC method of teaching is detailed in Table 5 and Figure 3. Both group responses were positive regarding FC as a method of teaching. An average of 80% said that they understood the topic well due to FC method and it increased their interest in it. Almost 75% of students agreed that group activities helped them in learning and they were well prepared for class. More than 80% of students liked how faculties communicated and directed discussion during FC method teaching. Students agreed that faculties motivated them during FC teaching. Around 78% of students agreed that they were curious about topics and FC method encouraged them to participate in the discussion. More than 50% of students disagreed or were neutral that FC method was time-consuming for learning. Students enjoyed learning through this method which helped them think critically. Overall FC method helped them enhance their learning of the subject.

**Table 5:** Feedback Questionnaire With Response Based on Likert Scale

No.	Questionnaire	Strongly Agree	Agree	Neutral	Disagree	Strongly Disagree
1	I understood the topic very well due to flipped classroom method of teaching.	32	33	12	3	2
2	Flipped classroom teaching increased my interest in Biochemistry.	33	34	9	4	2
3	Flipped classroom will cause higher retention of knowledge than routine didactic lectures for me.	32	29	16	2	3
4	I was able to learn through group activity in the class.	29	33	12	4	4
5	Due to flipped classroom method of teaching, I was usually well‑prepared for class.	29	32	16	3	2
6	The post-test made sense to me; I understood its purpose.	35	27	15	3	2
7	I felt encouraged to participate in pre-class online assignments.	22	38	17	1	4
8	The material given to me for flipped classroom method was useful.	26	35	15	4	2
9	The teacher treated us with respect.	47	22	8	2	3
10	The teacher effectively directed and stimulated discussion.	30	38	9	3	2
11	The teacher effectively encouraged us to ask questions and give answers.	35	25	12	7	3
12	I was curious to understand the topic due to post-test.	34	31	11	3	3
13	Learning through “Flipped Classroom” is time-consuming.	11	16	20	16	19
14	Flipped classroom method encouraged my active participation.	27	37	11	2	5
15	Flipped classroom method encouraged communication between us and teacher.	35	34	9	2	2

## Discussion

There have been tremendous changes that are coming up in relation to medical teaching. There are various teaching methods that are being employed for better learning outcomes. The current trend for learning is from a teacher-centered approach to a learner-centered active participation approach [7-9]. Effectiveness of the FC method against the TDC method was accessed in this study. It was found that learning skills and developing critical thinking through FC method were more effective and the acceptance ratio of students was better for the same. FC method model was welcomed by students. Feedback by students clearly summarized that they liked this new method of learning. Findings were consistent with previous studies that the FC method fostered students’ abilities in analyzing and solving clinical problems, thus improving the higher level of cognitive abilities [9,10].

There are few possible explanations for such strong liking by students. Traditional didactic lectures are faculty-centered where students remain mostly passive except for a few minutes of interactivity. They are told what to learn and nothing more making them passive recipients of knowledge. While in FC students come to center stage. This method is learner-centered. Students are supposed to engage among their peers in this method and provide their reflection on what is learned for the specific learning topics [11]. Pre-session resources shared among students encourage them to participate in the learning process and motivate them to study during pre-session through them or through searches on the web [12]. Information shared through resources in smaller blocks helps them grasp the essence of learning objectives very effectively. Taking small sips of knowledge compared to a single large bolus is always better for students. During the session period using small group discussion helps them recall easily and this helps in proper reinforcement of topics to be learned. Pre-session, which normally extends for around a week helps them effectively do time management. They are in charge of what to do with the learning resources provided. Having flexibility in learning makes students prepare thoroughly for the subject. Using current generation resources like the web, videos, etc., helps students feel at home [13,14].

Compared to TDC where there is only minor teacher-student interaction, FC method not only promotes teacher-student interaction but also student-student interaction. This may contribute to promoting teamwork ability, which is an essential ability in patient management for medical students [15]. In FC method, by nature, there is more interaction between teacher and students. This also helps faculty get proper feedback from students and improve upon learning of students [16]. As FC method is advantageous over TDC method in learning, there was a large chunk of students who felt that FC method is time-consuming and difficult. This may be due to group discussion being an integral part of the FC method, which may bring mental pressure along with it as reported in other studies [13,17]. It was observed that those students who used pre-session resources effectively, took part in discussion confidently without any pressure.

## Conclusions

FC, a novel method of interactive teaching, can be very beneficial in medical education. This method enhances the learning among medical students significantly. The perception of students toward FC teaching method was very positive. FC teaching method helps students grasp subjects to be learned at a faster pace and in a more effective manner.

This approach may help students develop critical thinking and medical skills effectively. If implemented along with regular medical teaching may improve students’ performance and enhance skills in examinations. The study required extensive participation of faculties during sessions. Applying the same method for a complete syllabus may require thorough planning and careful implementation. The present study was conducted on a small cohort of participants. So, with larger sample size studies, FC learning method will have a much stronger foothold as one of the better learning tools for medical studies. By modifying syllabus design, its management and evaluation according to FC method can achieve desired goals.
